# Differentiation and Function of Follicular CD8 T Cells During Human Immunodeficiency Virus Infection

**DOI:** 10.3389/fimmu.2018.01095

**Published:** 2018-05-22

**Authors:** Minglu Xiao, Xiangyu Chen, Ran He, Lilin Ye

**Affiliations:** ^1^Institute of Immunology, Third Military Medical University, Chongqing, China; ^2^Department of Immunology, School of Basic Medicine, Huazhong University of Science and Technology, Wuhan, China

**Keywords:** follicular CD8 T cells, B-cell follicles, human immunodeficiency virus infections, human immunodeficiency virus reservoir, CXCR5^+^CD8 T cells

## Abstract

The combination antiretroviral therapeutic (cART) regime effectively suppresses human immunodeficiency virus (HIV) replication and prevents progression to acquired immunodeficiency diseases. However, cART is not a cure, and viral rebound will occur immediately after treatment is interrupted largely due to the long-term presence of an HIV reservoir that is composed of latently infected target cells that maintain a quiescent state or persistently produce infectious viruses. CD4 T cells that reside in B-cell follicles within lymphoid tissues, called follicular helper T cells (TFH), have been identified as a major HIV reservoir. Due to their specialized anatomical structure, HIV-specific CD8 T cells are largely insulated from this TFH reservoir. It is increasingly clear that the elimination of TFH reservoirs is a key step toward a functional cure for HIV infection. Recently, several studies have suggested that a fraction of HIV-specific CD8 T cells can differentiate into a CXCR5-expressing subset, which are able to migrate into B-cell follicles and inhibit viral replication. In this review, we discuss the differentiation and functions of this newly identified CD8 T-cell subset and propose potential strategies for purging TFH HIV reservoirs by utilizing this unique population.

## Introduction

Human immunodeficiency virus (HIV)-specific CD8 T cells play an important role in suppressing HIV replication (1–5). The onset of HIV-specific CD8 T cell responses is concomitant with a reduction in plasma viremia (6–8). The rapidity and magnitude of HIV-specific CD8 T-cell responses correlate inversely with set-point viremia in hyperacutely infected patients (9). However, diminished HIV-specific CD8 T cell responses are accompanied by disease progression (10). Furthermore, elite controllers of HIV infection exhibit specific major histocompatibility complex (MHC) class I alleles and a wide spectrum of HIV-specific CD8 T-cell responses (11–15). Additionally, CD8 T cell-specific epitope mutants emerge to evade CD8 T-cell recognition during HIV infection (16, 17). Most direct evidence comes from rhesus macaques with chronic simian immunodeficiency virus (SIV) infection, in which transient CD8 T cell depletion resulted in a substantial increase in plasma viremia, while the subsequent replenishment of CD8 T cells led to a reduction in viremia (18–20). Despite the importance of HIV-specific CD8 T cells, they are not capable of fully eliminating HIV-infected target cells, mainly CD4 T cells. A wide variety of extrinsic and intrinsic factors are required to cripple HIV-specific CD8 T-cell mediated inhibition of HIV replication. One key factor lies in the functional exhaustion of HIV-specific CD8 T cells due to persistent T-cell receptor (TCR) stimulation and inhibitory microenvironments (21–23). Additionally, recent progress has been made to realize that HIV preferentially targets TFH cells in B-cell follicles for both long-term latent infection and the persistent production of infectious viral particles (24–28), and the majority of HIV-specific CD8 T cells are excluded from B-cell follicles (29–31). Therefore, the exhaustion of HIV-specific CD8 T cells and the anatomical separation of latently infected TFH cells and HIV-specific CD8 T cells might represent two primary barriers for HIV-specific CD8 T cells to eradicate HIV infection. Combination antiretroviral therapeutic (cART) is extremely effective at decreasing viremia to an undetected level (32–34); however, the viremia rebounds soon after the cessation of treatment (35–37). These facts further suggest that exhausted HIV-specific CD8 T cells cannot efficiently inhibit residual viral replication in the presence of effective cART treatment. However, a small fraction of CD8 T cells has been discovered to appear in B cell follicles in HIV infection as early as 1980 and 1990s (38–43). In 2007, Quigley et al. also reported that early effector memory CXCR5^+^CD8 T cells infiltrated into B cell follicles in human tonsil (44). Until recently, several groups reported a novel subset of exhausted HIV-specific CD8 T cells expressing CXCR5 and capable of migrating to B-cell follicles during HIV/SIV infection that rekindled interest in the filed (45–54). In this review, we focus on understanding the properties of HIV-specific CXCR5-expressing follicular cytotoxic cells and propose strategies for the functional cure of HIV infection by combining cART and CXCR5^+^CD8 T cells.

## Immune Exhaustion of Virus-Specific CD8 T Cells During Chronic HIV Infection

In response to an acute viral infection, virus-specific CD8 T cells recognize viral peptide–MHC class I complexes presented on the surface of antigen-presenting cells and subsequently become activated by signals transduced from TCR complexes and co-stimulatory receptors (55–57). The activated CD8 T cells in turn undergo dramatic proliferation and differentiate into effector CD8 T cells that are capable of efficiently clearing virally infected target cells by secreting anti-viral cytokines, such as TNF-α and INF-γ, as well as cytotoxic molecules, including perforin and granzymes. In the case of mouse lymphocytic choromeningitis virus (LCMV)-Armstrong and human influenza infection, and in response to smallpox and yellow fever vaccines, a large number of effector CD8 T cells with potent anti-viral functions eventually eradicate infectious viral particles within 8–10 days (58–60). Consistent with the resolution of viral infection and inflammation, the majority (>90%) of virus-specific effector CD8 T cells die of apoptosis, while a small fraction of these effector cells will survive and progressively differentiate into memory CD8 T cells (61–63). Memory CD8 T cells possess a stem cell-like property, being able to maintain themselves long-term through antigen-independent self-renewal driven by the cytokines interleukin-7 and IL-15 (59, 64). Most importantly, quiescent memory CD8 T cells largely preserve the epigenetic modification features of genes associated with effector functions that are developed at the effector stage, allowing these cells to rapidly exert multiple effector functions and efficiently clear invaded viruses soon after re-infections occur (65–67).

In contrast to acute viral infection, the continuous stimulation by persistent viral antigens due to unresolved chronic viral infection leads to a distinct differentiated state of activated virus-specific CD8 T cells termed immune exhaustion (68–71). Distinct from memory CD8 T cells, exhausted CD8 T cells exhibit several unique features, including, but not limited to, reduced cell proliferation potential upon re-stimulation, enhanced turnover rate due to being more prone to apoptosis, programmed and hierarchal loss of the ability to secrete cytokines and release cytotoxic granule components, prolonged and enhanced expression of an array of inhibitory receptors, altered epigenetic and metabolic signatures, and a failure to further convert to traditional memory CD8 T cells (72–76). The exhaustion of CD8 T cells was first discovered in a mouse model of chronic infection with LCMV and later on confirmed in various chronic viral infections in human, such as HIV and the hepatitis C and B viruses (21, 68, 77–80).

Similar to chronic LCMV infection in mice, chronic HIV infection does not clonally delete HIV-specific CD8^+^ T cells; instead, these cells also undergo a progressive and hierarchical loss of effector functions and display enhanced expression of a set of inhibitory receptors, such as programmed cell death-1 (PD-1), cytotoxic T-lymphocyte-associated protein 4 (CTLA-4), lymphocyte activation gene 3, and T cell immunoglobulin domain and mucin domain 3 (Tim-3), and a failure to differentiate into classical memory CD8 T cells evidenced by elevated apoptosis, diminished proliferation potential, and a rapid loss of CD127 expression (81–90). Moreover, at the genome-wide transcriptome level, similarities have been observed between exhausted LCMV-specific CD8 T cells and HIV-specific CD8 T cells (88, 91). The antigen load appears to be a critical cause that drives the development of these shared transcriptional signatures associated with CD8 T cell exhaustion in both chronic LCMV and HIV infection (23, 69, 92). The durable exposure to persistent antigen stimulation profoundly impacts the intrinsic epigenetic program and alters the expression mode of key transcriptional factors, such as T-bet, Eomes, TCF-1, Batf, and Id2-E2A, in exhausted LCMV- and HIV-specific CD8 T cells (23, 45, 72, 91, 93). The co-expression of inhibitory molecules, such as PD-1, CTLA4, and Tim-3, further promotes the extent of CD8 T cell exhaustion (22, 94–96). Additionally, the lack of optimal CD4 T cell help, at least partially mediated by IL-21 secreted from this population, represents another important factor for CD8 T cell exhaustion in both chronic LCMV and HIV infection (97–102). Furthermore, regulatory T cells (Tregs) and myeloid-derived suppressor cells (MDSCs) may further corroborate the progress of CD8 T cell exhaustion (103–107). The general similar characteristics between exhausted LCMV- and HIV-specific CD8 T cells highlight the great value of murine LCMV chronic infection as an informative experimental system to explore and reveal novel aspects of CD8 T-cell immunity in chronic HIV infection, even though murine LCMV infection is not an ideal model for HIV virology.

Although exhausted CD8 T cells are unable to differentiate into classical memory T cells, they are also not all terminally differentiated cells, which is supported by the consequences of the partial rescue of proliferative potential and effector function of exhausted CD8 T cells by targeting the PD-L1/PD-1 inhibitory pathway with an antibody blockade both *in vivo* (mouse chronic LCMV infection and rhesus macaque chronic SIV infection) and *in vitro* (co-culturing PD-L1 blockade antibodies with HIV-specific exhausted CD8 T cells) (108–110).

Furthermore, at the population level, exhausted CD8 T cells are not functionally inert and still maintain the critical ability to suppress viral replication during chronic LCMV and HIV infection (16–19, 111). The non-terminal differentiation state and partially preserved effector function of exhausted CD8 T cells provide precious opportunities for therapeutically targeting and reinvigorating exhausted CD8 T cells, which can possibly lead to the efficient control of chronic viral infection.

## Differentiation of the Follicular CXCR5-Expressing CD8 T-Cell Subset During HIV Infection

Although exhausted, virus-specific CD8 T cells preserve a certain ability to mediate an imperative suppression of viral replication in both chronic LCMV and HIV infection (3, 112–114). Given that the majority of virus-specific CD8 T cells are functionally exhausted, it is of great interest to investigate whether the exhausted CD8^+^ T cell pool contains a specific subset that are responsible for effectively keeping viral replication in check during chronic viral infection. Our recent study has found that during mouse chronic infection with the LCMV-Cl13 strain, but not acute infection with the LCMV-Armstrong strain, a unique subset of exhausted CD8 T cells expressing the chemokine receptor CXCR5 was differentiated (45). These virus-specific CXCR5^+^CD8 T cells possess the ability to migrate into B-cell follicles. Furthermore, CXCR5^+^CD8 T cells express lower levels of inhibitory receptors, such as PD-1, 2B4, and Tim-3, than their CXCR5^−^ counterparts, and accordingly, these cells demonstrate more potent cytotoxicity than the CXCR5^−^ subset. The Id2/E2A axis was found to play an important role in the generation of this subset. Specifically, E2A promotes the generation of this population while Id2 antagonizes this effect. In patients with chronic HIV infection, a virus-specific CXCR5^+^CD8 T cell subset was also identified in blood and lymph nodes, and the number of HIV-specific CXCR5^+^CD8 T cells inversely correlated with the viral load in blood. Similar to the scenario in chronic LCMV infection, HIV-specific CXCR5^+^CD8 T cells also show up in the follicular zone (45). Furthermore, HIV-specific CXCR5^+^CD8 T cells exhibit a reduction in Id2 expression compared to HIV-specific CXCR5^−^CD8 T cells. These similar characteristics of CXCR5^+^CD8 T cells during both chronic LCMV and HIV infection indicate that the differentiation of this unique subset might represent a common mechanism for defense against chronic viral infection.

Several other groups have also reported CXCR5^+^CD8 T cell populations during chronic LCMV infection, SIV and HIV infection. In chronic SIV and HIV infection, these reports uniformly demonstrated the follicular localization of CXCR5^+^CD8 T cells in lymphoid tissues (46, 47, 49, 53, 115, 116). The follicular location may depend on CXCR5 expression (117). However, in LCMV-Cl13 infection in mice, Im et al. found that the majority of these cells were localized in the T-cell zone (52), while we reported that these cells preferentially localized to the B-cell zone (45). This divergence remains an important issue to be further clarified and a possible explanation may be that Im et al. used antibody recognizing TCF-1 to stain CXCR5^+^CD8 T cells. As TCF-1 is also highly expressed in T-cell zone residing naïve and memory T cells (118, 119), which may potentially cause false positive. Intra-vital multi-photon confocal microscopy represents a reliable tool to visualize the dynamics of follicular-residing lymphocytes in a real-time pattern, which may provide more solid evidence as to the exact locations of virus-specific CXCR5^+^CD8 T cells in lymphoid tissues during chronic viral infection. Furthermore, both studies found that CXCR5^+^CD8 T cells preserved a better proliferative potential than CXCR5^−^CD8 T cells (45, 52). We also defined the continuous conversion of CXCR5^+^CD8 T cells into CXCR5^−^CD8 T cells during LCMV chronic infection in mice, which was likely driven by elevated Id2 expression in CXCR5^+^CD8 T cells (45). The replenishment of this population critically depends on new emigrants from the thymus (45). It is worthwhile to investigate whether these features also hold true in chronic SIV and HIV infection, which can be determined by using non-human primate models and a bone marrow–liver–thymus humanized mouse model, respectively.

**Figure 1:**
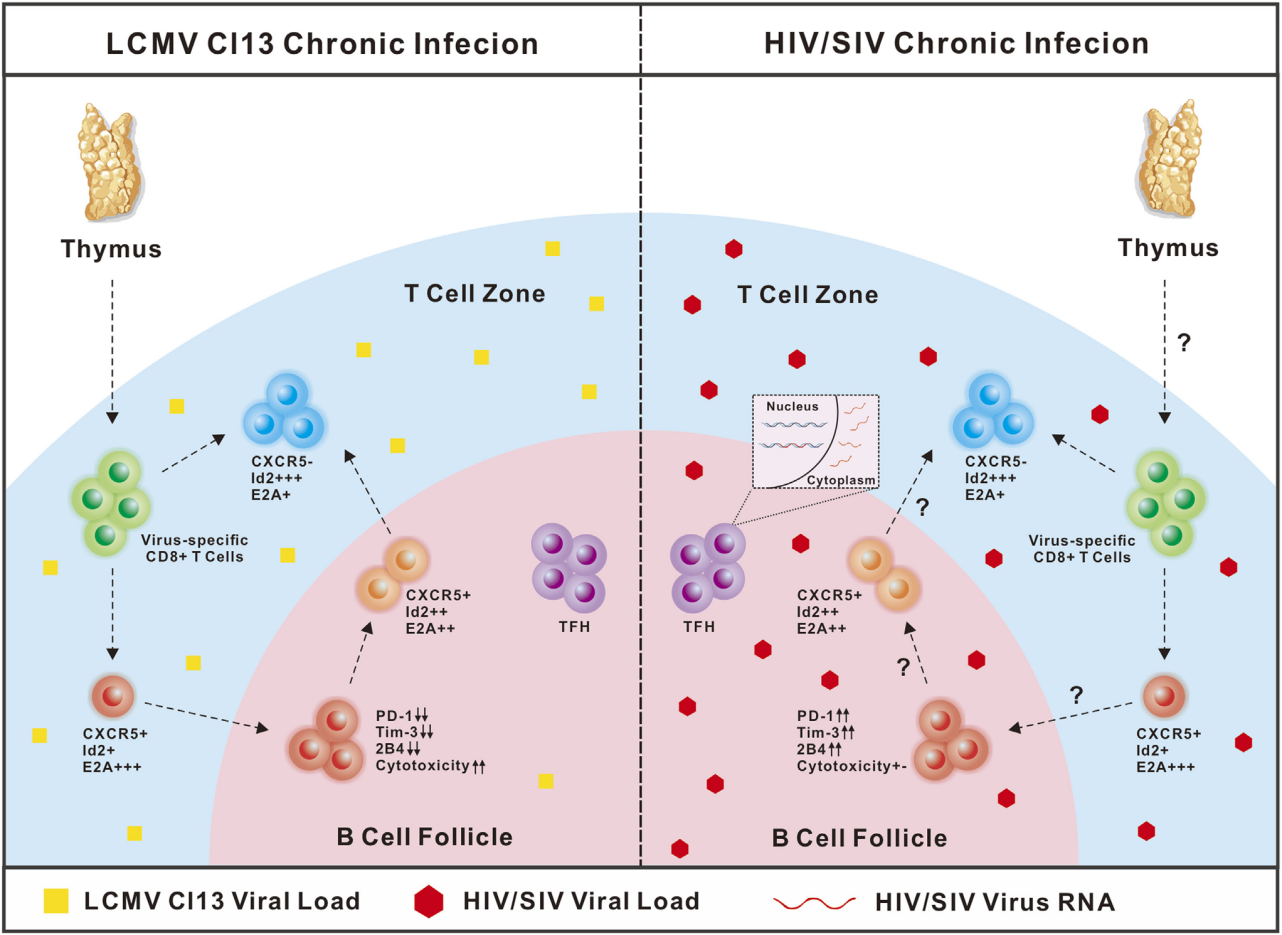
Comparison of CXCR5^+^CD8 T cells in lymphocytic choromeningitis virus (LCMV)-Cl13 and human immunodeficiency virus (HIV) infection. In chronic LCMV-Cl13 infection, viruses seldom infect B-cell follicles, thus B-cell follicles function as a sanctuary for CXCR5^+^CD8 T cells to prevent rapid exhaustion. In contrast, HIV virus preferentially targets TFH cells in B-cell follicles for productive and latent infection, thus accumulating high antigen loads in B-cell follicles may drive more severe exhaustion of CXCR5^+^CD8 T cells.

It should be noted that in chronic LCMV-Cl13 infection in mice, viruses seldom infect cells residing in B-cell follicles, while in chronic SIV and HIV infection, viruses predominantly and productively infect follicle-residing TFH cells (25, 120–122). Therefore, in LCMV-Cl13 infection, the antigen loads and inhibitory microenvironment in B-cell follicles are relatively friendly toward virus-specific CXCR5^+^CD8 T cells, and B-cell follicles may function as a sanctuary for virus-specific CXCR5^+^CD8 T cells to prevent the rapid loss of their number and effector functions. In contrast, in chronic SIV and HIV infection, viral replication is more concentrated in TFH cells in B-cell follicles (29, 120, 123). Therefore, the high antigen loads in B-cell follicles may drive the more severe exhaustion of follicle-residing HIV-specific CXCR5^+^CD8 T cells. The enhanced strength and duration of TCR stimulation from high antigen loads cause the rapid loss of these exhausted cells by apoptosis, which may partially explain the scarcity of this subset in B-cell follicles in chronic SIV and HIV infection. PD-1 is a central mediator that negatively regulates the exhaustion of virus-specific CD8 T cells (81, 124). In chronic LCMV infection, virus-specific CXCR5^+^CD8 T cells were found to express relatively lower PD-1 levels compared to virus-specific CXCR5^−^CD8 T cells (45). In contrast, during chronic HIV infection, HIV-specific CXCR5^+^CD8 T cells expressed higher levels of PD-1 than their CXCR5^−^ counterparts (47). This divergence in PD-1 expression in virus-specific CXCR5^+^CD8 T cells during chronic LCMV and HIV infection might be largely attributed to the different antigen load levels in B-cell follicles during chronic LCMV and HIV infection (Figure 1) (Table 1).

**Table 1:** The similarities and differences of CXCR5^+^CD8 T cells in lymphocytic choromeningitis virus (LCMV)-CI13 and human immunodeficiency virus (HIV)/simian immunodeficiency virus (SIV) infection.

Similarities	The number is inversely correlated with viral load in blood The subset was identified in blood and lymphoid organs Preserve a better proliferative potential than CXCR5^−^CD8 T cells Transcription factor feature: Id2 ↓, E2A ↑ Possess the ability to migrate into B cell follicles	

	**Lymphocytic choromeningitis virus**	**SIV/HIV**
Differences	Viruses seldom infect cells residing in B-cell follicles, providing a friendly microenvironment (low antigen load) for CXCR5^+^CD8 T cells	Viruses predominantly infect follicle-residing TFH cells leading to a nasty microenvironment (high antigen load) for CXCR5^+^CD8 T cells

	Lower programmed cell death-1 (PD-1) expression than CXCR5^−^ counterparts	Higher PD-1 expression than CXCR5^−^ counterparts

	More potent cytotoxicity than CXCR5^−^ counterparts (e.g. IFN-γ, TNF-α, and degranulation)	Controversial issue: enhanced or comparable effector functions relative to CXCR5^−^CD8 T cells?

## The Functionality of Follicular CXCR5-Expressing CD8 T-Cell Subset During HIV Infection

In LCMV-Cl13 infection, compared to CXCR5^−^CD8 T cells, virus-specific CXCR5^+^CD8 T cells exhibit elevated effector cytokine expression, including IFN-γ and TNF-α, in response to antigen stimulation. They also display enhanced degranulation. Consistent with these characteristics, these cells are more efficient at killing target cells *in vivo* than the CXCR5^−^ counterparts. Furthermore, when adoptively transferred to CD8-deficient recipients chronically infected with LCMV-Cl13, virus-specific CXCR5^+^CD8 T cells, but not CXCR5^−^CD8 T cells, can effectively inhibit viral replication in recipients (45). Together, these results demonstrate that virus-specific CXCR5^+^CD8 T cells preserve better effector functions than CXCR5^−^CD8 T cells in suppressing chronic viral infection. However, there are conflicting results regarding the functionality of CXCR5^+^CD8 T cells in chronic HIV infection. Several reports have shown that compared to CXCR5^−^CD8 T cells, CXCR5^+^CD8 T cells show an increase in the production of IFN-γ, TNF-α, and perforin; enhanced degranulation and cytolytic activities (45, 46, 53, 115). In contrast, a recent study demonstrated the comparable production of cytolytic proteins between HIV-specific CXCR5^+^CD8 T cells and CXCR5^−^CD8 T cells in lymphoid tissues from patients with chronic infection, but much lower than that of HIV-specific CD8 T cells in blood (50). It should be noted that HIV-specific CXCR5^+^CD8 T cells are still exhausted cells. Similar to LCMV-specific CXCR5^+^CD8 T cells, these cells are most likely heterogeneous, consisting of newly recruited cells (less exhausted due to the short exposure time to antigens) from thymic outputs and past generated cells (more exhausted due to the concentrated viral replication in B-cell follicles). Whole body viral loads and disease progression might potentially influence the ratio between newly and past generated HIV-specific CXCR5^+^CD8 T cells. Interestingly, Miles et al. reported that the majority of follicular CD8 T cells are regulatory CD8 T cells with the expression of CD44 and CXCR5. This regulatory subset expresses less perforin and high level of Tim-3 to inhibit IL-21 production by TFH cells and impairs GC function in SIV and *ex vivo* HIV infection (49). However, whether these regulatory CD8 T cells are SIV- or HIV-specific awaits further investigation. Furthermore, we may not rule out the possibility that certain subset of antigen-specific CD8 T cells become *de novo* CXCR5-expressing cells and be included in the analysis in responding to antigen stimulation. Furthermore, the more exhausted state of CXCR5^+^CD8 T cells from more concentrated viral antigens in B-cell follicles may also explain their lower cytolytic activities when compared to total HIV-specific CD8 T cells in blood. Next, it is interesting to directly compare the functional capacity of HIV-specific CXCR5^+^CD8 T cells from lymphoid tissues and blood (50).

Although there are conflicting results regarding the expression of inhibitory molecules and their functional capacities, it is a consensus that the number of HIV- or SIV-specific CXCR5^+^CD8 T cells inversely correlates with plasma viremia and disease progression (31, 45, 48, 54, 116), highlighting a critical functional role of this subset in viral control during chronic SIV or HIV infection. Given the possible downregulated effector functions and cytolytic activities, this important characteristic of CXCR5^+^CD8 T cells might be largely attributed to their non-terminal differentiation state and better-retained proliferative potential. Indeed, HIV-specific CXCR5^+^CD8 T cells express less Id2 (promoting terminal differentiation) and higher TCF-1 (promoting memory differentiation and proliferative potential) than CXCR5^−^CD8 T cells (45, 47, 51, 125). In a chronic LCMV-Cl13 infection model, overexpressing Id2 or ablating TCF-1 leads to the impaired generation of virus-specific CXCR5^+^CD8 T cells and accordingly to increased viral loads (45, 47, 52). Moreover, virus-specific CXCR5^+^CD8 T cells, but not CXCR5^−^CD8 T cells, respond to the PD-1–PD-L1 pathway blockade and increase clonal expansion (45, 52). In chronic HIV infection, memory-like HIV-specific CXCR5^+^CD8 T cells may persist longer than their CXCR5^−^ counterparts at population levels and continuously kill virus-infected cells. By contrast, because B-cell follicle-residing TFH cells are major virus producers compared to other CD4 T cell types in the T cell zone in HIV infection (24, 120), it is reasonable to infer that HIV-specific CXCR5^+^CD8 T cells, but not CXCR5^−^CD8 T cells, have chances to come into contact with and kill these target cells. Therefore, HIV-specific CXCR5^+^CD8 T cells primarily rely on their memory-like properties and unique anatomical location for their critical control of viral replication in the context of chronic HIV infection.

## Strategies for Employing CXCR5^+^CD8 T Cells to Purge HIV Reservoirs in B-Cell Follicles

It has been well-documented that virus-specific CD8 T cells are required for the elimination of HIV reservoirs (3, 112, 126). Accumulating evidence has demonstrated that TFH cells in B-cell follicles of lymphoid tissue serve a major HIV reservoir, as viruses preferentially target TFH populations for productive and latent infection (25, 26). Taking into account that a limited number and exhausted state of HIV- or SIV-specific CXCR5^+^CD8 T cells were present in B-cell follicles, this unique strategy largely protects these viruses from the attacks mediated by virus-specific CD8 T cells. Indeed, in elite controllers from chronic SIV infection, SIV-specific CD8 T cells can effectively control viral replication at extra-follicular sites; however, the majority of these cells fail to migrate to B-cell follicles to clear SIV-producing TFH cells (19, 30, 31). In ART-treated, aviremic non-human primates and patients, lymph node PD-1^+^TFH populations also serve as a major reservoir for active and persistent viral transcription (28). Thus, HIV reservoirs harbored in TFH cell populations in lymph node B-cell follicles represent a major obstacle for a functional cure for HIV infection. To this end, the appearance of a large number of HIV-specific CXCR5^+^CD8 T cells equipped with potent cytotoxic functions is a prerequisite for effectively eliminating TFH reservoirs under cART treatment. Additionally, CXCR5^+^CD8 T cells are not stable and will eventually convert into CXCR5^−^CD8 T cells, which will exit B-cell follicles (45). Therefore, we speculate that the rational design of strategies for a functional cure for HIV infection will rely on the following three important aspects: (1) enhanced virus-specific CXCR5^+^CD8 T cell differentiation, (2) preserved lineage stability, and (3) functional reinvigoration.

**Figure 2:**
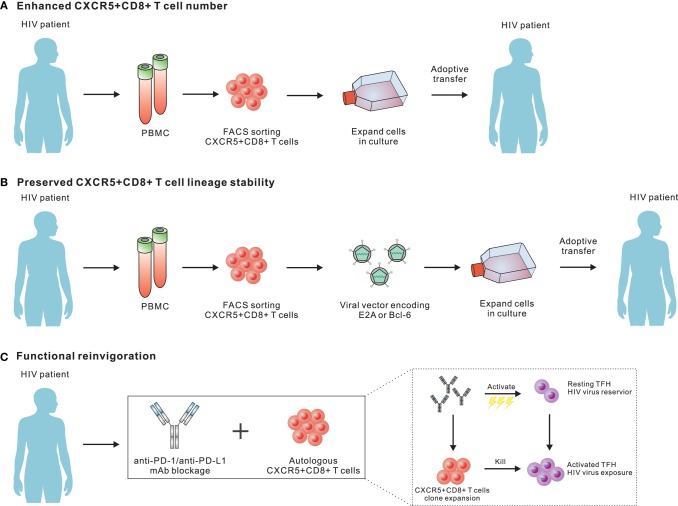
Potential strategies for employing CXCR5^+^CD8 T cells to purge human immunodeficiency virus (HIV) reservoirs in B cell follicles. **(A)** Adoptive transfer of *in vitro* expanded endogenous CXCR5^+^CD8 T cells from blood. As CXCR5^+^CD8 T cells from peripheral blood will further differentiate into CXCR5^−^CD8 T cells upon antigen re-stimulation, the development of *in vitro* culturing conditions optimal for both expanding and preserving the migratory and functional characteristics of CXCR5^+^CD8 T cells should be a focus for future investigations. **(B)** Transfer genetically modified virus-specific CD8 T cells over-expressing transcriptional factors that promote the differentiation and lineage stabilization of CXCR5^+^CD8 T cells. **(C)** Programmed cell death-1 pathway blockade may effectively expand transferred virus-specific CXCR5^+^CD8 T cells and boost the effector functions.

In chronic LCMV-Cl13 infection, we have shown a greater therapeutic potential for LCMV-specific CXCR5^+^CD8 T cells than the CXCR5^−^ subset upon adoptive transfer to chronically infected mice, as well as synergistic effects that reduce the viral load when combined with anti-PD-L1 treatment (45). In an SIV model or HIV patients, it is also worth testing the efficacy of this combination for suppressing HIV replication and latency in TFH cells in non-human primates or patients under ART treatment. Virus-specific CXCR5^+^CD8 T cells, but not CXCR5^−^CD8 T cells, are PD-1 pathway blockade responders (52). In this regard, PD-1 blockade antibodies can effectively expand transferred virus-specific CXCR5^+^CD8 T cells and boost the effector functions of these cells. Velu et al. demonstrated that during chronic SIV infection, PD-1 blockade resulted in rapid expansion of virus-specific CD8 T cells with improved functionality (127). However, the reduction of plasma viral load seemed not to be that impressive, which may be due to a very limited number of CXCR5^+^CD8^+^ T cells *in situ*. Therefore, the combination of PD-1 blockade with adoptive transfer of large number of virus-specific CXCR5^+^CD8 T cells may further improve the control of viral replication. Furthermore, the PD-1 pathway blockade may also have partial effects on TFH cells, which express a high abundance of PD-1. The activation of TFH cells latently infected with viruses by the PD-1 antibody blockade may enhance the transcription of viral genes, which may increase antigenic exposure for cytotoxic killing due to the transfer of virus-specific CXCR5^+^CD8 T cells. In addition to the adoptive transfer of *in vitro* expanded endogenous CXCR5^+^CD8 T cells from blood, it is also possible to transfer genetically modified virus-specific CD8 T cells over-expressing transcriptional factors that promote the differentiation and lineage stabilization of CXCR5^+^CD8 T cells, such as E2A and Bcl-6 (Figure 2). Besides, adoptive transfer of antiviral chimeric antigen receptor (CAR) T cells co-expressing the follicular homing chemokine receptor CXCR5 could potently suppress SIV replication *in vivo* (128). In addition to adoptive transfer, therapeutic vaccination plus the PD-1 pathway blockade may also boost the differentiation and functional rescue of virus-specific CXCR5^+^CD8 T cells (129, 130). Furthermore, a recent study has demonstrated that a novel IL-15 agonist ALT-803 could activate and direct SIV-specific CD8 T cells into B cell follicles *via* upregulation of CXCR5 (131). Thus, the combination of IL-15 agonist and strategies mentioned above may offer a new immunotherapeutic agent for purging HIV reservoirs in B-cell follicles.

## Conclusion

Based on the phenotypic, anatomic, and functional characterization of virus-specific CXCR5^+^CD8 T cells in chronic viral infection, this subset has drawn immense attention and many new findings have been learned allowing us to better understand its features. Published data have firmly established the notion that this population is localized in B-cell follicles and plays a critical role in repressing viral load during chronic HIV infection. However, many important questions remain unanswered regarding the basic biology of this unique subset. For example, little is known regarding the origin and early fate commitment of this subset. Additionally, we barely know the cytokine milieu that is involved in the differentiation of this subset. Understand the characteristics of these cells will facilitate the optimization of *in vitro* culture conditions for the efficient expansion of these cells for therapeutic purposes.

It is also important to investigate whether and how these cells kill TFH cells that are actively transcribing viral RNAs in B-cell follicles during HIV infection. It is also of great interest to examine whether this population is required for the elimination of latently infected TFH cells after “shock and kill” intervention. Furthermore, we need to develop various immune strategies, such as vaccination, that can be utilized to efficiently induce and stabilize this population. Provided that the major reservoir is harbored in B-cell follicle TFH cells, and CXCR5^+^CD8 T cells are the sole virus-specific population that has the chance and ability to gain access to these reservoirs, understanding the molecular mechanisms underlying the differentiation, migration, and function of this unique subset will definitely provide important insights that will allow us to harness this population for a functional cure against HIV infection.

## Author Contributions

MX, RH, and LY wrote and edited the manuscript. XC designed the figures.

## Conflict of Interest Statement

The authors declare that the research was conducted in the absence of any commercial or financial relationships that could be construed as a potential conflict of interest.
